# Twittering About Research: A Case Study of the World’s First Twitter Poster Competition

**DOI:** 10.12688/f1000research.6992.3

**Published:** 2016-06-20

**Authors:** Edward P. Randviir, Samuel M. Illingworth, Matthew J. Baker, Matthew Cude, Craig E. Banks

**Affiliations:** 1 Faculty of Science and Engineering, School of Science and the Environment, Division of Chemistry and Environmental Science, Manchester Metropolitan University, Manchester, UK; 2 WestCHEM, Department of Pure and Applied Chemistry, Technology and Innovation Centre, University of Strathclyde, Glasgow, UK; 3 Royal Society of Chemistry, Cambridge, UK

**Keywords:** Twitter, Research, Poster, Competition, Engagement, Communication, Chemistry, Conference

## Abstract

The Royal Society of Chemistry held, to our knowledge, the world’s first Twitter conference at 9am on February 5 ^th^, 2015. The conference was a Twitter-only conference, allowing researchers to upload academic posters as tweets, replacing a physical meeting. This paper reports the details of the event and discusses the outcomes, such as the potential for the use of social media to enhance scientific communication at conferences. In particular, the present work argues that social media outlets such as Twitter broaden audiences, speed up communication, and force clearer and more concise descriptions of a researcher’s work. The benefits of poster presentations are also discussed in terms of potential knowledge exchange and networking. This paper serves as a proof-of-concept approach for improving both the public opinion of the poster, and the enhancement of the poster through an innovative online format that some may feel more comfortable with, compared to face-to-face communication.

## Introduction

An academic conference should be a symposium where academics can report, share, discuss their work, and exchange ideas through a variety of different communication methods. A typical academic conference may consist of several oral presentations, including those from keynote or plenary speakers, in addition to a number of workshops, which offer a more interactive method of delivery. There is also the research poster, a somewhat maligned and misunderstood entity that in some instances feels like an afterthought. However, it could be argued that if knowledge exchange is the fundamental purpose of a conference, which it is, then posters and workshops are far more valuable than oral presentations ( Rowe & Ilic, 2009); in which case, are posters being unfairly discredited?

The “all eyes on one” style of oral presentations is extremely limiting in terms of opportunities for the speaker to interact personally with members of the audience. Even in the post-talk questions, time constraints mean that not all questions can be asked, whilst some participants may be unable to comfortably relay their points to the author in that particular environment. Aside from this, the less intimidating nature of poster sessions may be preferable to a larger percentage of researchers, which might explain why the poster presentation saw large increases in the 1990s ( Moule *et al*., 1998).

A poster session is an extended period of academic knowledge exchange. Exhibitors normally stand by their poster and explain their research and findings to passing delegates, inspiring some form of discussion as a dialogue or perhaps in a group. Therefore, it makes sense poster sessions should provide more frequent opportunities for academics to exchange knowledge and create networks. The format of a poster session should theoretically allow for open, informal, and comfortable academic discussion regarding the work presented. Many researchers will have experienced instances of such academic exchanges taking place; yet it is not a form of communication that has been formally investigated in any great detail, but for contributions from Dubois Betty (1985) and Shalom (1993), who independently suggest that the poster presentation was a genre struggling for definition some 20 years ago; the feeling is unfortunately similar within scientific disciplines today. However, it has also been shown that students presenting posters on sensitive topics found that the format of the poster session put them more at ease ( Rush *et al*., 1995). Such an observation would suggest that the poster acts as a message board and focal point for presenters, with sensitive topics such as sexuality made easier to discuss by using posters as a facilitator. This facilitatory role can be extended to other less taboo-orientated subjects and, in principle, the poster could help to facilitate learning amongst researchers, especially those in the early stages of their careers who may be less confident when presenting their research, compared to other, more experienced colleagues.

Despite many efforts by academics to report good poster guidelines (see e.g. Erren & Bourne, 2007; Hess *et al*., 2009; Moore *et al*., 2001; Shelledy, 2004; Taggart & Arslanian, 2000), the ideal poster presentation is often absent from poster sessions. Many posters are either poorly designed, or simply pinned to boards and left to stagnate, leaving any observant or enthusiastic researchers with unanswered questions. Even if a poster manages to attract a delegate, the content must be written in a concise, clear, and jargon-free manner to inspire intrigue. Poor written communication can be as detrimental to the message as the oral communication blunders brought about by an ill-prepared delivery.

It therefore comes as no surprise that some organisations have attempted to reimagine the poster. One specific example of this comes from the European Geosciences Union (EGU), who use a concept called PICO (Presenting Interactive COntent) to diversify the knowledge exchange process. The general idea of PICO is for researchers to orally advertise their work in a two-minute flash presentation, in order to encourage the audience to later join them at interactive touchscreen slides, where they can engage with the author personally, in a format similar to the traditional poster session ( European Geosciences Union, 2015). Such a form of engagement will no doubt enhance the learning and knowledge exchange experience for the researcher.

With any conference, there are always academics wishing to participate, but who are unable to because of travel and funding restrictions. For some researchers, these restrictions can be detrimental for the dissemination of their research, and can ultimately have a negative effect on their career progression. To combat this, some organisations, like the American Geophysical Union (AGU), have piloted a virtual poster showcase, encouraging researchers to participate at conferences virtually through a digital link. This obviates the requirement for travel, and therefore extra funding for travel purposes. Furthermore, posters are becoming an ever more acceptable route into publication, *via* academic journals such as in *F1000Research*, which publishes posters and slides alongside more traditional articles, as a means of reference-worthy academic literature.

Another potential alternative is the use of social media to encourage poster engagement, and this route will form the focus of this paper. The ubiquity of social media is responsible for many of the social behaviours and patterns that have emerged as a result of online communication, and given the power of social media, it could potentially be harnessed to help ensure posters are more greatly discussed, thereby helping to improve ideas and knowledge exchange between academics. This paper presents findings from the world’s first Twitter poster conference, organised by the Royal Society of Chemistry, and discusses the potential impact of social media upon the academic poster.

## Materials and methods

### Conference organisation

The Analytical Science Twitter Poster Conference (ASTPC) was organised by the Royal Society of Chemistry (RSC) journals *Analyst*, *Analytical Methods* and *Journal of Analytical Atomic Spectroscopy (JAAS)*. The ASTPC took place from 9am on 5 ^th^ February 2015 to 9am on the 6 ^th^ February 2015, giving researchers a period of 24 hours to tweet pictures of their poster using the hashtag #RSCAnalyticalPoster. The aim of the ASTPC was to create an opportunity for participants to showcase their research, meet new people, share ideas and learn about some of the latest developments in different areas of analytical science. The conference was open to anyone working in any area of analytical science whose research topic was within the scope of *Analyst, Analytical Methods*, or *JAAS*.

### Data production

Participants were encouraged to tweet their work, and to be available to answer any questions that interested academics (or indeed members of the general public) might have about their research. There were also prizes for the best Twitter poster, as judged by the scientific committee, with remuneration in the form of an iPod and RSC book vouchers. Furthermore, unlike a regular conference that charges fees to participate, this event was entirely free, and had no registration process other than an email to the journal to verify identity. A scientific committee consisting of 12 academics associated with the RSC were heavily involved in asking questions, generating discussions, and judging posters. Further information regarding the event can be found on the journal’s official blog ( http://blogs.rsc.org/an/2014/12/19/rscanalyticalposter/). We have no way of knowing whether the competitive element skewed the participation of this conference, but feel that at most scientific conferences there are prizes awarded for the best posters, so in this regard assume that the competitive element does not increase nor decrease participation.

This study was carried out according to the British Educational Research Association’s (BERA) ethical guidelines for educational research, with all of the data in this study fully anonymised. All work was also carried out according to the terms of use as indicated by Twitter's policies.

### Measurement of Twitter activity

The participants that took part in the ASTPC are now assessed in terms of the number of tweets, area of the world from which the tweet was sent, total number of followers, and potential viewing audience for the tweets. All data is sourced from an online data collection program, available at http://www.followthehashtag.com. The data sample was taken over a period of 63 days, from 9am on 19 ^th^ December 2014 to 9am on 20 ^th^ February 2015. The data was collected from such an early date because this is when the initial announcement of the hashtag was made and promotion of the event began, however the vast majority of tweets were sent during the 24-hour window of the competition itself. Data collection stopped shortly after the prize winners were announced. Only tweets with the hashtag #RSCAnalyticalPoster were considered for the analysis, and so any figures reported here are most likely an underestimate, precluding any tweets for which the hashtag was absent. All reported times are in Greenwich Meantime.

## Results and discussion

Raw data for 'Twittering About Research: A Case Study of the World's First Twitter Poster Competition' Data have been de-identified. Click here for additional data file. Copyright: © 2016 Randviir EP et al. 2016 Data associated with the article are available under the terms of the Creative Commons Zero "No rights reserved" data waiver (CC0 1.0 Public domain dedication).

**Figure 1.**
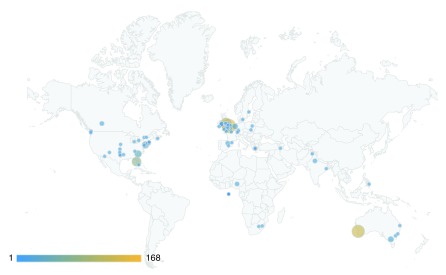
World map depicting the locations of participants in the ASTPC. Yellow points indicate multiple contributions, whilst blue data points indicate singular or near-singular contributions. Reproduced from data reports obtained from the website http://www.followthehashtag.com.

Figure 1 depicts a world map with the locations of persons that contributed to the ASTPC. Over 80 posters were submitted from Argentina, Australia, Brazil, Canada, Ireland, Italy, Mauritius, the UK, and the USA, with the highest number of contributors coming from European countries. From the diversity shown in Figure 1, it can be inferred that social media can be used to improve the accessibility of the poster by making it freely accessible across the world in a matter of minutes. This further presents opportunities for researchers to exchange comments in the form of tweets, a format that is designed to be both clear and concise. Such communiqués encourage researchers to think more directly about their research, as they must communicate their point in 140 characters or less. This concise form of communication could help both students and academics to communicate more effectively, particularly students who sometimes struggle to differentiate between description and analysis ( Chanock, 2000).

Table 1 presents the statistics that were published following the ASTPC. During the designated time period, over 1700 tweets were sent with the hashtag #RSCAnalyticalPoster, originating from 378 different contributors. Each participant contributed 4.59 tweets on average to the discussion, with the total number of followers for each person that tweeted amounting to over 380,000. On average, every poster potentially received in excess of 4200 views from several areas across the world, based upon the total impressions divided by the number of contributors (see Figure 1). This figure assumes that every impression was knowingly observed, but this is obviously unlikely.

**Table 1.** Data obtained from the ASTPC.

Total tweets	1,734
Total audience (sum of followers)	381.233
Contributors (no. of unique Twitter users)	378
Measured time	63 days (19/12/15 to 20/02/15)
Total impressions*	1,594,269
Impressions per audience	4.18
Tweets per contributor	4.59
Tweets per day	27.5

**Total tweets** - the total number of tweets which included #RSCAnalyticalPoster, this includes retweets. **Total audience** - the number of people who may have seen #RSCAnalyticalPoster in their Twitter feed. Calculated using the sum of followers from each contributor. **Contributors** - number of unique Twitter accounts that used #RSCAnalyticalPoster. **Total impressions** - the sum of contributor followers multiplied by the number tweets in which a contributor used #RSCAnalyticalPoster. *Impressions are defined as the number of times a tweet is “served” in a Twitter timeline or search result.

**Figure 2.**
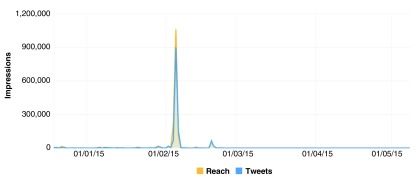
Number of Twitter impressions as a function of time, expressed both as tweets and reach ^+^; reproduced from data reports obtained from the website http://www.followthehashtag.com. ^+^Reach is defined as the number of observers of the tweet, based upon the number of followers of the user and the followers of any participants, responses, or retweets.

Figure 2 displays a tweet and reach timeline that illustrates the frequency of activity across the 63 days of data collection. It is evident that there are two major zones of activity, as one would largely expect: the first when the conference was officially held, and the second when the prize winners were announced. The biggest reach and number of tweets was observed during the main event, followed by the prize announcements. Figure 2 also demonstrates that the majority of the tweets took place during the ASTPC itself, relating to the scientific posters rather than to advertising and promotion of the conference.

**Figure 3.**
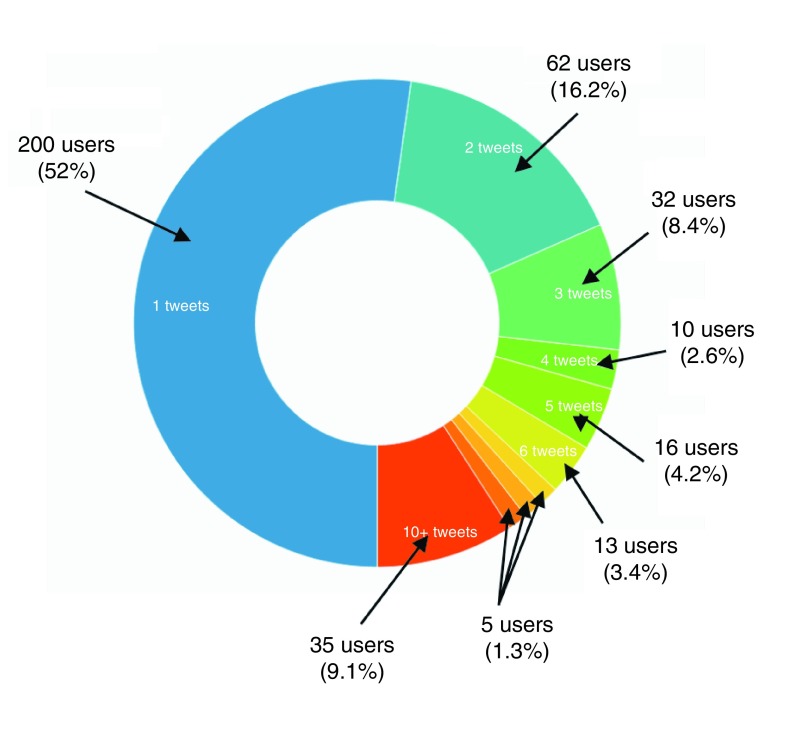
Illustration of the number of tweets sent by individuals; blue = 1 tweet; red = 10+ tweets. Reproduced from data reports obtained from the website http://www.followthehashtag.com.

Given the nature of a Twitter discussion, it is perhaps more useful to present data relating to the number of contributions that users made as a whole, rather than as an average. Figure 3 depicts the individual contributions by author, and it is apparent that over half of the tweeters made only one contribution to the competition (200 users). More encouragingly, over 20% of contributors tweeted five or more times, and almost 10% of the contributors tweeted more than ten times. Indeed, the latter statistic infers that at least some useful exchanges were being made, even if it is difficult to gauge from such data how successful the exchanges may have been. The overall reach of each individual is difficult to estimate from such a dataset. One contributor may have contributed ten tweets to the discussion that has 300 people contributing, for example, yet only have ten followers, giving an overall reach value of around 310 people. Conversely, one person may have 1000 followers yet only contribute one tweet, yet their reach would be around 1300 people for one tweet alone. Therefore, it is fair to assume that Twitter can have a larger impact if the user has more followers, regardless of the number of contributions. This is one area that will be useful for further investigation, insofar as number of exchanges within one single conversation, number of tweets in that exchange, the relation of the interactions to the work presented (or indeed if it was just friendly chat), and the possibility of other collaborators engaging in dialogue over social media. This is currently beyond the scope of this work, but will be the subject of future investigations.

**Figure 4.**
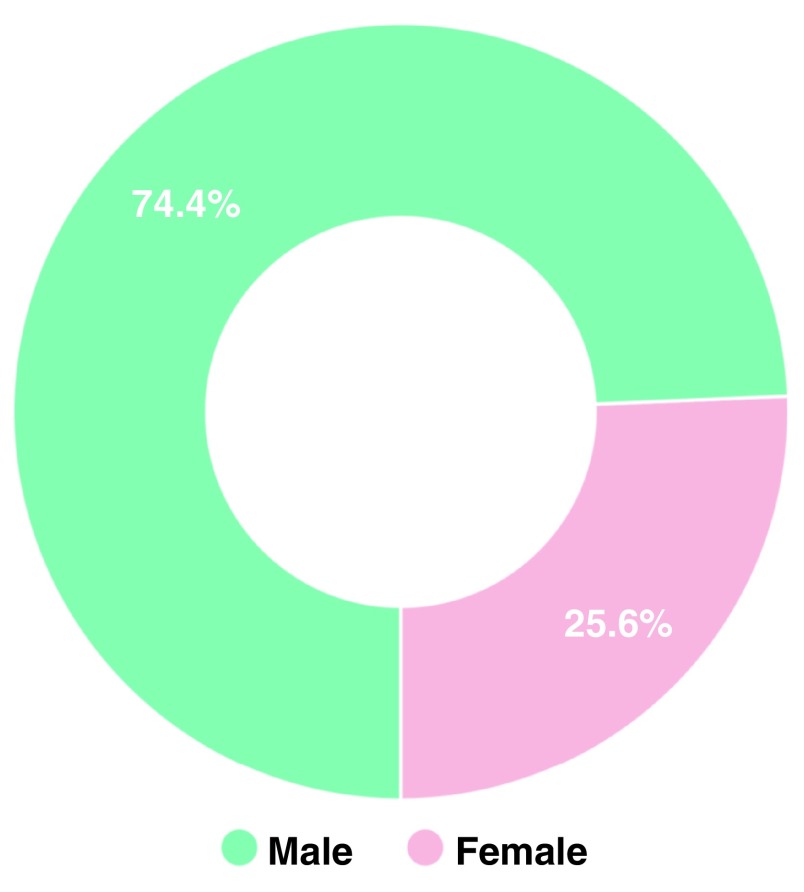
Gender distribution of the ASTPC contributions; reproduced from data reports obtained from the website http://www.followthehashtag.com.

Another important piece of information relates to the gender distribution at the Twitter conference. According to the RSC membership department, 27.7% of their members are female and 72.3% male, representing an uneven distribution of members by gender. Figure 4 displays the contributions of the ASTPC by gender, with 25.6% of contributions made by females and 74.4% by males. The fact that there is no significant difference between the RSC’s overall membership and the contributors at this event shows that the social media format is not conducive to stimulation of more or less average contributions based upon the gender of the participant. The level of participation in terms of registrants was different to this, however, as 59.6% of the registrants were female. Therefore, whilst a large proportion of females were willing to engage with the competition (a significantly higher proportion than would be expected based on the RSC membership), the discussions appeared to be dominated by male contributors.

The ASTPC was organised as a free event to encourage the sharing and exchange of knowledge through the use of social media. This pilot scheme saw a potential Twitter audience of over a quarter of a million people, demonstrating that posters can quite easily be shared using Twitter, to potentially reach thousands of times more people than they could at even the largest of international scientific conferences. Every day millions of people across the world access Twitter, new Twitter connections are being made, and opinion and discussion is stimulated as a result, while the introduction of hashtags has only served to group discussions together and augment the potential reach of a niche discussion. Even without an organised hashtag or event, a poster can have a larger potential audience than it would at a conference, where the audience will, at the very most, be a few hundred people. The number of useful exchanges between participants is less easy to enumerate, as one cannot quantify the level of interaction between academics in a given poster session and compare it to the number of tweets. A face-to-face conversation about a poster that is in front of two researchers will no doubt be more fulfilling in terms of knowledge exchange, because the conversation is not limited to a few characters. In the Twitter conference, there was an average of 4.5 tweets per person, suggesting that the level of academic discussion was somewhat limited. However, this is not to say that knowledge was not exchanged, but simply that the discussion part of the ASTPC may have been shorter than that of a standard poster session at a conference. This does not account for exchanges that may have been made in private, via emails or direct messaging facilities on Twitter.

As a concept, the Twitter poster conference has some definite advantages over a more traditional poster format, with the data analysed in this study supporting the notion that it is an extremely useful way of broadening the reach and potential audience of a poster. Another advantage is the ease of knowledge exchange for those who lack the confidence or interpersonal skills required for efficient face-to-face communications. It is also apparent that Twitter can decrease the cost of the poster to the researcher because it does not need to be carried as supplementary luggage during air travel; it also avoids potentially exorbitant printing fees at conferences for those who have lost or previously been unable to print their poster. Furthermore, the carbon footprint of a Twitter-only conference is extremely low (unquantified), whereas an international conference will exhibit a substantial carbon footprint, mainly due to air travel. Research by MMU (unpublished report, Jonathan Davies and Professor Callum Thomas) has recently found that an international conference of 178 delegates resulted in the equivalent of 177 tonnes of CO _2_ being produced, the majority of which came from the 1.25 million kilometres of air travel required for delegates to travel to the conference.

The nature of Twitter means that more in-depth forms of communication are limited through online exchanges, which could be seen as a disadvantage of the format. However, after the initial exchanges the delegate has the opportunity to extend any interactions further. This can be achieved by the exchange of emails, phone numbers, and Skype IDs for example, or in private messaging facilities over Twitter, meaning that more in-depth chats about the research in question can still be facilitated. Future Twitter conferences should incorporate a feedback device for participants (presenters or otherwise) to understand what benefits the format has to the participant and whether they would participate again, with or without the element of competition. The lack of interpersonal communication is disadvantageous, but should not detract researchers from a Twitter conference. It could be used alongside the traditional poster session, or as a separate entity of its own if a researcher is unable, or prefers not, to travel. Given Twitter’s recent foray into video streaming, the concept could also be adapted to include oral communications, in which researchers could tweet short video vignettes of their work, or even use Periscope to live-stream the entire presentation. The Twitter format could also potentially be used as a hybrid with the PICO concept discussed in the introduction.

In specific relation to the RSC, the results presented here indicate that whilst Twitter could be used as a tool to address the gender inequality, more needs to be done to encourage female participants to participate in the active Twitter discussions.

## Conclusions

The world’s first Twitter conference could be considered a success in terms of potential audience, ease of knowledge exchange and lack of travel requirement. The conference reached out to many researchers across the world, and created an opportunity for participants to share their work not only with academics, but also with other interested parties such as writers, industries, friends and family, and even policy makers. Over 80 posters were tweeted with the hashtag #RSCAnalyticalPoster, reaching an audience potentially as large as 375,000 people, and the format of a Twitter poster conference has the potential to allow for research to be shared more quickly and cheaply, and in a more environmentally friendly manner. Despite some potential issues relating to prolonged exchanges, there is no doubt that the hybridisation of the academic conference and social media is something that could and should be seen more regularly in the future. We expect the use of social media to significantly expand scientific conferences due to the advantages identified above, and also to be utilised alongside conferences where physical participation occurs. The benefits of social media can help researchers organise their poster viewings at large conferences, for example future ACS conferences, helping to potentially improve the poster session experience for all participating researchers. Such an improvement in engagement will enhance scientific communication and knowledge exchange, ultimately leading to more successful conferences. Future investigations will focus specifically upon detailed Twitter interactions in such an academic context and investigate whether the interactions can be classified as “meaningful”, in order to establish the true impact of a social media only conference.

## Data availability

The data referenced by this article are under copyright with the following copyright statement: Copyright: © 2016 Randviir EP et al.

Data associated with the article are available under the terms of the Creative Commons Zero "No rights reserved" data waiver (CC0 1.0 Public domain dedication). 

F1000Research: Dataset 1. Raw data for ‘Twittering About Research: A Case Study of the World’s First Twitter Poster Competition’., 10.5256/f1000research.6992.d101516 ( Randviir *et al.,* 2015).

All data is also available publically by searching for the hashtag “#RSCAnalyticalPoster” on FollowtheHashtag.
