# Supplementary material for: Twittering About Research: A Case Study of the World’s First Twitter Poster Competition
Source: F1000Res. 2016 Jun 20;4:798. Originally published 2015 Sep 14. [Version 3] doi: 10.12688/f1000research.6992.3 (PMC4706062; doi:10.12688/f1000research.6992.3)
Supplement: Peer review history

# Peer review: Referee response for version 2

**DOI:** 10.5256/f1000research.8186.r11764

**Published:** 2016-01-07

**Authors:** Graham Scott

**Affiliations:** 1 School of Biological, Biomedical, and Environmental Sciences, University of Hull, Hull, UK

I am content that the authors have addressed the points made.

I have read this submission. I believe that I have an appropriate level of expertise to confirm that it is of an acceptable scientific standard.

# Peer review: Referee response for version 2

**DOI:** 10.5256/f1000research.8186.r11775

**Published:** 2015-12-31

**Authors:** Michael Bales

**Affiliations:** 1 Weill Cornell Medical College, New York, NY, USA

I have read this submission. I believe that I have an appropriate level of expertise to confirm that it is of an acceptable scientific standard.

# Peer review: Referee response for version 1

**DOI:** 10.5256/f1000research.7529.r11359

**Published:** 2015-12-15

**Authors:** Michael Bales

**Affiliations:** 1 Weill Cornell Medical College, New York, NY, USA

The authors describe an academic conference and poster competition that occurred online in February 2015. Researchers were invited to share their work via Twitter in the form of an academic poster during a specified window of time on the day of the conference. The authors compare and contrast the online conference with poster sessions at traditional academic conferences, identifying several advantages and disadvantages.

The article is well-written and serves as a good contribution to a growing body of work focusing on the changing modalities of scholarship and academic collaboration.

Several minor comments:

In the description for Table 1, the authors state that "on average, every poster potentially received in excess of 4500 views..." So that this caption can stand on its own, it would be helpful to clarify why the word "potentially" is used in this case.

In Figure 2 there one line for "Reach" and another for "Tweets", but the Y-axis is labeled "Impressions". Given that readers may be expecting a second Y-axis, the caption for Figure 2 should be sufficient to explain these three concepts.

I have read this submission. I believe that I have an appropriate level of expertise to confirm that it is of an acceptable scientific standard.

# Response

**Published:** 2015-12-17

**Authors:** Craig Banks

**Affiliations:** Manchester Metropolitan University, UK

We thank the reviewer for their comments and have endeavored to respond appropriately.

- The word "potentially" is used because we feel that there is no way that each poster had over 4200 views - the figure derives from the number of impressions divided by the total contributors, and assumes that every impression is knowingly observed. We have included this in the manuscript.
- The caption in Figure 2 has been defined more appropriately.

# Peer review: Referee response for version 1

**DOI:** 10.5256/f1000research.7529.r11113

**Published:** 2015-12-10

**Authors:** Durhane Wong-Rieger

**Affiliations:** 1 Canadian Organization for Rare Disorders, Toronto, ON, Canada

The concept and successful execution of this "experiment" makes it worth publishing. The data collected supports the premise that sharing a poster through a dedicated # will attract participants. However, some of the other concerns raised about the limitations of more traditional research poster sessions do not seem to be addressed. It is not clear why this was conducted as a competition and whether there would have been the same level of participation without this element. So, the level of engagement might have been biased by a factor not usual in a poster session. It would have been very useful to have a "qualitative" content analysis of the tweets, or at least a sample of tweets to understand what was exchanged in the messages. This would add to understanding of the potential value of a Twitter-fest for scientific exchange. At least an analysis of the scope or "build" of discussion would be useful. How many exchanges were two, three or four exchanges? How many exchanges were builds on a core comment or additions to an initial exchange?

It may also have been beneficial to have a discussion as to the implications of the countries participating.   

The conclusions of success based on "ease of knowledge exchange, lack of travel requirement, and reduction in carbon footprint" are somewhat questionable, given that these are the qualities of social media (especially Twitter) and so do not reflect on the success or value of this activity.

It may have been useful to solicit some feedback from participants, including the presenters, as to how they felt about the experience, the quality of the exchange, and the interest in doing again.

I have read this submission. I believe that I have an appropriate level of expertise to confirm that it is of an acceptable scientific standard.

# Response

**Published:** 2015-12-14

**Authors:** Craig Banks

**Affiliations:** Manchester Metropolitan University, UK

We thank the reviewer for providing an insightful review that will not only help improve the manuscript, but will also affect our future work.

- While we understand the reviewer’s thought regarding the competitive element, we feel that most scientific conferences of this nature offer prizes for the best posters. With this in mind, we feel that the competitive element is no different to other conferences and assume that the participation does not change based upon this. We have noted this in the experimental section.

- The referee makes an excellent point regarding the nature of exchanges. We feel that with the information we have collected, we cannot extract enough quality data to report within this paper, but future work will focus more on a detailed analysis of Twitter interactions and develop a model for the level of meaning in each interaction. We have pointed this out within the updated manuscript in the conclusion, and have also discussed in the text that we feel further detail regarding exchanges would be useful.
- We agree that a feedback method should have been incorporated and this will be included in future works. The discussion section has been changed slightly to reflect the fact that this was an opportunity missed.
- We have removed the conclusion regarding lack or travel and carbon footprint, but remain optimistic that Twitter improves ease of knowledge exchange, if not directly then indirectly.

# Peer review: Referee response for version 1

**DOI:** 10.5256/f1000research.7529.r10299

**Published:** 2015-09-21

**Authors:** Graham Scott

**Affiliations:** 1 School of Biological, Biomedical, and Environmental Sciences, University of Hull, Hull, UK

This is an interesting paper that describes and evaluates a Twitter poster conference. The authors have clearly highlighted the potential reach that Twitter provides scientists wishing to engage others with their work and the ways in which Twitter as a medium has distinct advantages over traditional face to face interaction. In doing so this paper provides a useful starting point for a discussion about the use of social media as a conference format.

The authors acknowledge the limitations of twitter (e.g. limited ability to have an in depth discussion) but from the presented data they are unable to discuss this in any detail. In my opinion this is an area that should be investigated further. As an initial foray into the field this is a (very necessary) descriptive rather than investigative piece. In order that I might fully evaluate the value of the model I would have like to have more information about the broader themes of the tweets themselves – what proportion of them asked/answered a relevant question? How many were simply an indication of approval or an acknowledgement of participation in the virtual meeting etc. I don’t think that this information needs to be added to the current paper (it makes a valuable initial contribution) but I would encourage the authors to follow this work with such an analysis if that is possible.

There is one minor change that I think would increase the initial readability of the paper. On first reading I found myself confused about the nature of the conference, perhaps revealing a prejudice based on prior experience and pre-conception I assumed that the Twitter poster conference was an adjunct to a physical meeting rather than a stand alone event. It may help readers to avoid this mistake if the authors explained that more clearly in the abstract?

I have read this submission. I believe that I have an appropriate level of expertise to confirm that it is of an acceptable scientific standard.

# Response

**Published:** 2015-12-14

**Authors:** Craig Banks

**Affiliations:** Manchester Metropolitan University, UK

We thank the referee for their insightful comments and feel that they are of benefit to the manuscript both now and in the future.

- We are in agreement that this area should be investigated further, and have included some information as to how we will extend this work in the future, specifically relating to the nature of Twitter exchanges and in developing a level of meaning based upon the information within exchanges.
- The referee makes a valid point regarding the ambiguity surrounded this event as a standalone conference. The abstract has been changed to reflect the nature of the conference to make this less confusing.
